# Preclinical obesity curriculum: audit, implementation, and evaluation

**DOI:** 10.1186/s12909-024-05606-9

**Published:** 2024-06-07

**Authors:** Amber Olson, Rosanna Watowicz, Eileen Seeholzer, Katherine Lyons, W. Scott Butsch, Colleen Croniger

**Affiliations:** 1 Case Western Reserve University School of Medicine, Cleveland, OH USA; 2 Department of Nutrition, Case Western Reserve University School of Medicine, Cleveland, OH USA; 3 Adult Weight Loss Surgery & Weight Management Center, MetroHealth System, Cleveland, OH USA; 4 Bariatric and Metabolic Institute, Cleveland Clinic, Cleveland, OH USA

**Keywords:** Obesity, Curriculum development, Competency-based education, Obesity attitudes and knowledge

## Abstract

**Background:**

This study aimed to (1) evaluate the current status of obesity education at Case Western Reserve University School of Medicine (CWRU) (2), introduce a comprehensive first-year curriculum on obesity, and (3) assess the impact of the curriculum on self-reported attitudes and knowledge regarding obesity among first-year medical students.

**Methods:**

The preclinical curriculum at CWRU was reviewed to determine the degree of coverage of Obesity Medicine Education Collaborative (OMEC) competencies for healthcare professionals, and recommendations were provided for revising the curriculum to better adhere to these evidence-based competencies. A survey on obesity attitudes and knowledge was given before and after the implementation of the new curriculum to measure intervention-related changes. Changes in obesity attitudes and knowledge were compared (1) before and after the intervention for the class of 2025 and (2) after the intervention for the class of 2025 to a historical cohort that did not receive the intervention.

**Results:**

Among the 27 competencies examined in the audit, 55% were unmet and 41% were partially met. Of 186 first-year medical students (M1s), 29 (16%) completed the baseline survey and 26 (14%) completed the post-intervention survey. Following the intervention, there was a notable improvement in attitudes and knowledge regarding obesity. Specifically, there was a significant decrease in the belief that obesity is caused by poor personal choices, and knowledge of obesity in fourteen out of fifteen areas showed significant improvement from pre- to post-intervention. Additionally, obesity attitudes and knowledge were significantly better post-intervention when compared to the historical cohort.

**Conclusions:**

The improvements made to the preclinical curriculum through this project improved obesity attitudes and knowledge among first-year medical students. This method provides a practical approach for evaluating and enhancing obesity education in medical school curricula.

**Supplementary Information:**

The online version contains supplementary material available at 10.1186/s12909-024-05606-9.

## Background

Obesity is a complex chronic disease that impacts over 41.9% of the US adult population, with higher rates seen in minority groups [1]. Its prevalence is on the rise, with estimate medical costs related to obesity in the United States reaching $173 billion in 2019 [2]. Obesity’s detrimental health effects extend to every organ, making it one of the largest contributors to preventable, noncommunicable diseases like heart disease, stroke, and cancer [1].

Despite obesity’s prevalence, cost, and adverse health effects, medical students lack education about obesity and training in obesity management. In 2007, the Association of American Medical Colleges (AAMC) published a Contemporary Issues in Medicine Report VIII entitled *The Prevention and Treatment of Overweight and Obesity* and concluded that future physicians needed to be better prepared through their medical education to provide respective, effective care of patients with overweight and obesity [3]. Despite this recommendation, little progress has been made on improving obesity education [4, 5].

A 2020 study aiming to describe the state of obesity education in undergraduate medical education illustrates the need for obesity education reform in medical schools [6]. They found that only 10% of medical schools reported their students are very prepared to treat patients with obesity [7]. One-third of schools reported they had no obesity education program in place and no plans to develop one, and half of schools reported obesity education to be a low priority or not a priority at all [7]. The greatest barrier to incorporating obesity education was lack of room in the curriculum [7] Studies of obesity coverage in graduate medical education have shown that obesity is similarly neglected during residency training [8, 9].

The Obesity Medicine Education Collaborative (OMEC) competencies provided an opportunity (1) to compare the Case Western Reserve University School of Medicine (CWRU) curriculum with accepted standards for obesity education and (2) to implement reforms to better adhere to these competencies. Spearheaded in 2016 by the Obesity Medicine Association (OMA), The Obesity Society (TOS), and the American Society for Metabolic and Bariatric Surgery (ASMBS), the OMEC competencies were created as the first set of obesity-related competencies that are based on the Six Core Domain Competencies of the Accreditation Council for Graduate Medical Education (ACGME) that are used in both undergraduate and graduate medical education programs [10].

This study had three objectives. First, this study assessed the state of obesity education at CWRU by evaluating adherence to OMEC competencies through a comprehensive audit. Second, this study implemented a first-year obesity preclinical curriculum to better adhere to OMEC competencies. Third, a survey tool was administered both before and after the implementation of the curriculum to evaluate the self-reported change of attitudes toward and knowledge of obesity among first-year medical students.

## Methods

This study was conducted at Case Western Reserve University School of Medicine (CWRU) in Cleveland, OH and approved by the Case Western Reserve University Institutional Review Board. Informed consent was obtained from all participants in the study.

### Audit

CWRU’s current preclinical curriculum was systematically assessed to determine the coverage of OMEC competencies. The CWRU preclinical curriculum is divided into 8 blocks (Table 1). All components of each block—including lectures, multiple-choice questions, team-based learning sessions, Case Inquiry (IQ) sessions, end-of-week free response questions, anatomy and radiology sessions, histopathology sessions, doctoring seminars, communication workshops, and physical diagnosis sessions—were reviewed. Both required and elective components of the curriculum were reviewed. One member of the research team (A.O.) reviewed all components of the curriculum and made the determination about the level of coverage. The audit determined the coverage of 27 OMEC competencies among five core domains: patient care and procedural skills, medical knowledge, interpersonal and communication skills, professionalism, and system-based practice. A Likert scale was developed by the OMEC from 1 (no coverage) to 5 (full coverage), and this scale was used to rate the degree of implementation of each competency. The OMEC core domain entitled practice-based learning and improvement was not included in this audit, as it was determined that this core domain is not relevant to the preclinical curriculum at CWRU. If a competency scored 1, it was determined that the competency was unmet. If a competency scored 2–3, it was determined that the competency was partially met. If a competency scored 4–5, it was determined that the competency was met.

**Table 1:** CWRU school of medicine preclinical curriculum

Block	Components	Recommendations	Large Changes
(1) Becoming a Doctor	Population health, epidemiology, biostatistics, bioethics, health disparities	*13 recommendations*: usage of person-first language, obesity as a chronic disease, obesity prevalence and disparities, weight bias	
(2) The Human Blueprint	Endocrinology, reproduction, development, genetics, molecular biology, cancer biology	*29 recommendations*: usage of person-first language, impact of obesity on reproduction and pregnancy, relationship between obesity and type 2 diabetes, role of genetics in obesity, relationship between obesity and cancer	
(3) Food to Fuel	Gastrointestinal, nutrition, biochemistry	*52 recommendations*: usage of person-first language, obesity pathophysiology, nutritional concepts, relationship between obesity and dyslipidemia, hormones that affect hunger and satiety, relationship between obesity and NAFLD, clinical assessment of obesity, obesity treatment	Two, two-hour team-based learning sessions (TBLs) on obesity pathogenesis and treatment Nutrition concepts lecture Obesity assessment lecture Exam question on obesity pathogenesis and treatment
(4) Homeostasis	Cardiovascular, pulmonary, renal, cell physiology, pharmacology	*10 recommendations*: usage of person-first language, relationship between obesity and cardiovascular disease and hypertension, relationship between obesity and obstructive sleep apnea	
(5) Host Defense and Host Response	Immunology, microbiology, hematology, oncology, infectious diseases, rheumatology, dermatology, musculoskeletal	*9 recommendations*: usage of person-first language	
(6) Cognition, Sensation, and Movement	Neurology, mind		
(7) Structure	Gross anatomy, radiology, living anatomy, histopathology	*10 recommendations*: usage of person-first language, physical examination skills in patients with obesity, impact of obesity on medical imaging	Exam question on obesity and challenges in medical imaging
(8) Foundations of Clinical Medicine	Communication, physical diagnosis, clinical skills, ethics, professionalism, cultural competence, quality improvement, law and medicine, patient safety	*15 recommendations*: usage of person-first language, weight bias, obesity disparities and access to care, physical examination skills in patients with obesity	Standardized patient with obesity in motivational interviewing communication workshop

### New preclinical curriculum

Based on the results of the audit, evidence-based recommendations were made for the revision of current curricular content and the addition of new curricular content to better adhere to the OMEC competencies. Recommendation sheets were emailed to all block leaders in the CWRU curriculum. Meetings were held with each block leader to discuss the recommendation sheets in detail. The number and types of recommendations that were made can be viewed in Table 1.

Several large additions were made to the existing CWRU preclinical curriculum. Two, two-hour team-based learning sessions were held on November 17, 2021 (Obesity Pathogenesis) and January 19, 2022 (Obesity Treatment) for all first-year medical students. The sessions were facilitated by two obesity medicine physicians, a dietitian, a professor in nutrition, and a second-year medical student [11]. A standardized patient was also incorporated into a communication workshop for students to practice counseling patients with obesity. Each student engaged in a standardized patient encounter to practice motivational interviewing regarding lifestyle changes. Each student was then assessed on their performance and given feedback. The new curriculum began with the class of 2025, starting at the beginning of their M1 year.

### Survey

A questionnaire on attitudes toward and knowledge of obesity was administered before and after the implementation of the obesity curriculum to gauge intervention-related changes (see Additional File 1). After reviewing the existing literature on tools used to assess attitudes toward and knowledge of obesity, a 37-item Likert scale questionnaire was created. Twenty-two items inquired about attitudes toward obesity, based on the NEW Attitudes Scale [12]. Respondents were asked about their extent of agreement using a Likert scale from 1 (completely disagree) to 5 (completely agree). The section on obesity knowledge consisted of fifteen competencies, based on the Medical School Curriculum Benchmark Study [7]. Respondents were asked to rate their knowledge of each competency using a Likert scale from 1 (not at all knowledgeable) to 4 (very knowledgeable). The baseline questionnaire was administered via email by the CWRU Department of Nutrition in August 2021, which was the beginning of M1 for the class of 2025 and the beginning of M2 for the class of 2024. After the obesity curriculum was carried out during the 2021–2022 academic year, the post-intervention questionnaire was administered in August 2022, at the beginning of M2 for the class of 2025, to assess for changes in attitudes toward and knowledge of obesity after the implementation of the obesity medicine curriculum. Study data was collected and managed using REDCap electronic data capture tools hosted at Case Western Reserve University [13].

A composite self-perceived knowledge score (out of 60) was computed by taking the sum of the obesity knowledge questions. Because responses were anonymous, baseline questionnaire data could not be paired with post-questionnaire data. Because unpaired t-tests have been used in other studies to determine the effect of a medical education intervention when pairing was not available, we decided to use unpaired t-tests in this study to assess the short-term impact of our intervention [14]. Since a high number of hypothesis tests were being conducted, we decided *a priori* to use a conservative *p*-value of < 0.01 to establish statistical significance. The change in obesity attitudes and knowledge was compared (1) pre- to post-intervention for the class of 2025 and (2) post-intervention for the class of 2025 to a historical cohort without the intervention (*p*-value < 0.01). The pre- to post-intervention comparison gives a sense of where the new curriculum may have improved obesity attitudes and knowledge. Comparing the class of 2025 post-intervention to the historical comparison cohort at the same time point (class of 2024) provides stronger evidence for the impact of the new curriculum. Analysis was conducted with JASP.

## Results

### Audit

Of all 27 OMEC competencies studied in the audit (Table 2), 15 (55%) were unmet in the CWRU curriculum and 11 (41%) were partially met. Only 1 competency—obesity-related comorbidities—was met in the CWRU curriculum.

**Table 2:** Audit of CWRU School of Medicine Curriculum according to OMEC competencies

Competency	Unmet	Partially Met	Met
***Patient Care and Procedural Skills***			
Elicits comprehensive obesity-focused medical history.	✓		
Performs and documents a comprehensive physical examination for the assessment of obesity.		✓	
Effectively applies clinical reasoning skills when ordering and interpreting appropriate laboratory and diagnostic tests during the evaluation of patients with obesity.		✓	
Utilizes evidence-based models of health behavior change to assess patients’ readiness to change in order to effectively counsel patients for weight management.		✓	
Engages the patients and their support systems in shared decision making by incorporating their values and preferences in the development of a comprehensive personalized obesity management care plan.		✓	
***Medical Knowledge***			
Demonstrates knowledge of obesity epidemiology.		✓	
Demonstrates knowledge of energy homeostasis and weight regulation.	✓		
Demonstrates knowledge of anthropometric (body composition) measurements and clinical assessments of energy expenditure.	✓		
Demonstrates knowledge of the etiologies, mechanisms, and biology of obesity.	✓		
Demonstrates knowledge of obesity-related comorbidities and the corresponding benefits of body mass index (BMI) reduction.			✓
Applies knowledge of the principles of primary, secondary, and tertiary prevention of obesity to the development of a comprehensive, personalized obesity management care plan.		✓	
Applies knowledge of obesity treatment guidelines to the development of a comprehensive, personalized obesity management care plan.	✓		
Applies knowledge of using nutrition interventions to develop a comprehensive, personalized obesity management care plan.	✓		
Applies knowledge of using physical activity interventions to develop a comprehensive, personalized obesity management care plan.	✓		
Applies knowledge of using behavioral interventions to develop a comprehensive, personalized obesity management care plan.	✓		
Applies knowledge of using pharmacological treatments of obesity as part of a comprehensive, personalized obesity management care plan.	✓		
Applies knowledge of the surgical treatments of obesity as part of a comprehensive, personalized obesity management care plan.		✓	
Applies knowledge of emerging treatment modalities for obesity to the development of a comprehensive, personalized obesity management care plan.	✓		
***Interpersonal and Communication Skills***			
Uses appropriate language in verbal, nonverbal, and written communication that is non-biased, non-judgemental, respectful, and empathetic when communicating with patients with obesity.	✓		
Uses appropriate language in verbal, nonverbal, and written communication that is non-biased, non-judgemental, respectful, and empathetic when communicating about patients with obesity with colleagues within one’s profession and other members of the healthcare team.	✓		
Demonstrates awareness of different cultural views regarding perceptions of desired weight and preferred body shape when communicating with the patient, family, and other members of the healthcare team.		✓	
***Professionalism***			
Demonstrates ethical behavior and integrity when counseling patients and their families who are living with overweight or obesity.		✓	
Displays compassion and respect toward all patients and families who are living with overweight or obesity.		✓	
***Systems-Based Practice***			
Works collaboratively within an interdisciplinary team dedicated to obesity prevention and treatment strategies.	✓		
Advocates for policies that are respectful and free of weight bias.	✓		
Utilizes chronic disease treatment and prevention models to advance obesity intervention and prevention efforts within the clinical, community, and public policy domains.		✓	
Describes the costs of obesity intervention and prevention with regards to the individual, health care system, and community.	✓		

### Attitudes toward obesity

Of 186 M1s in the intervention cohort, 29 (16%) completed the baseline survey and 26 (14%) completed the post-intervention survey. Of 184 M2s in the historical comparison cohort, 51 (28%) completed the baseline survey. After this intervention, attitudes toward obesity improved. Mean scores for attitudes toward obesity both before and after the intervention, as well as compared to the historical comparison cohort are presented in Table 3. Notably, the attitude that obesity is caused by poor personal choices decreased significantly from baseline to post-intervention (2.86 vs. 1.89, *p* < 0.001), representing a decrease in stigma, and was significantly less than the historical comparison cohort (2.75, *p* < 0.001). Confidence in treating obesity increased significantly from baseline to post-intervention (2.66 vs. 3.58, *p* < 0.01) and was significantly greater than the historical comparison cohort (2.45, *p* < 0.001). Perceived self-efficacy in treating obesity also increased significantly from baseline to post-intervention (2.38 vs. 3.42, *p* < 0.001) and was significantly greater than the historical comparison cohort (2.45, *p* < 0.001). This intervention did not change students’ personal desires to counsel patients about weight management, nor did it change the students’ attitude regarding obesity as a disease. Surprisingly, students largely agreed that it is important to counsel patients about weight management and that obesity is a disease, even prior to the intervention.

**Table 3:** Attitudes toward obesity*

	Mean (SD) intervention pre-test (beginning of M1) (*n* = 29)	Mean (SD) intervention post-test (beginning of M2) (*n* = 26)	Mean (SD) historical control group (beginning of M2) (*n* = 51)	Pre-test vs. post-test *p*-value	Post-test vs. historical control *p*-value
Obesity is a disease.	4.17 (0.71)	4.39 (1.06)	4.22 (0.90)	0.38	0.47
Obesity is caused by poor personal choices.	2.86 (0.95)	1.89 (0.86)	2.75 (0.84)	< 0.001	< 0.001
On average, individuals with obesity have less willpower than individuals without obesity.	2.21 (1.05)	1.46 (0.58)	2.18 (0.91)	< 0.01**	< 0.001
On average, individuals with obesity are more lazy than individuals without obesity.	2.21 (1.01)	1.42 (0.57)	2.06 (0.88)	< 0.001**	< 0.01
On average, individuals with obesity are more emotional than individuals without obesity.	1.86 (0.69)	1.42 (0.64)	1.88 (0.79)	0.02	0.02
People can eat a healthy diet if they choose to do so.	2.41 (1.12)	2.42 (0.86)	3.22 (1.03)	0.97	< 0.01
Counseling about nutrition does not change behavior.	2.35 (0.67)	2.27 (0.67)	2.02 (0.65)	0.68	0.12
Patients are likely to follow an agreed-upon plan to increase their exercise.	2.72 (0.84)	3.23 (0.71)	2.96 (0.89)	0.02	0.19
Even if I counsel them, patients will continue their poor exercise habits.	2.59 (0.83)	2.58 (0.90)	2.65 (0.77)	0.97	0.72
Weight loss is the result of eating less and exercising more.	3.14 (1.30)	2.39 (1.02)	3.12 (1.13)	0.02**	< 0.01
It is usually sufficient to give a person brief, clear advice about weight management.	1.90 (0.90)	1.62 (0.70)	1.98 (0.81)	0.21	0.06
Weight management counseling takes too much time.	2.10 (0.62)	1.85 (0.97)	2.22 (0.73)	0.25**	0.06
I think patients with obesity are motivated to change their lifestyle.	3.28 (0.75)	4.04 (0.77)	3.41 (0.88)	< 0.001	< 0.01
I believe that my patients will follow through with a weight management program.	2.86 (0.69)	3.50 (0.95)	3.14 (0.83)	< 0.01	0.09
I believe patients can maintain weight loss.	3.72 (0.75)	3.92 (0.85)	3.67 (0.79)	0.36	0.19
Patients know the health risks associated with obesity.	2.76 (1.12)	3.54 (1.03)	3.29 (1.08)	< 0.01	0.34
Patients take their weight seriously.	3.45 (1.06)	3.89 (0.82)	3.61 (0.67)	0.09**	0.12
I feel confident treating patients with obesity.	2.66 (1.08)	3.58 (0.86)	2.45 (0.86)	< 0.01	< 0.001
I feel effective in helping patients with obesity manage their weight.	2.38 (0.94)	3.42 (0.95)	2.45 (0.86)	< 0.001	< 0.001
I think treating patients with obesity is not worth the time.	1.31 (0.54)	1.31 (0.68)	1.41 (0.67)	0.99	0.52
If a patient has obesity, I feel uncomfortable discussing their weight.	2.35 (0.94)	2.08 (0.89)	2.51 (1.03)	0.28	0.07
I have a personal desire to counsel patients about weight management.	3.21 (1.11)	3.12 (1.14)	3.35 (0.96)	0.77	0.34

*using a Likert scale from 1 (completely disagree) to 5 (completely agree)

**Welch’s test used instead of Student’s test

### Knowledge of obesity

**Fig. 1:**
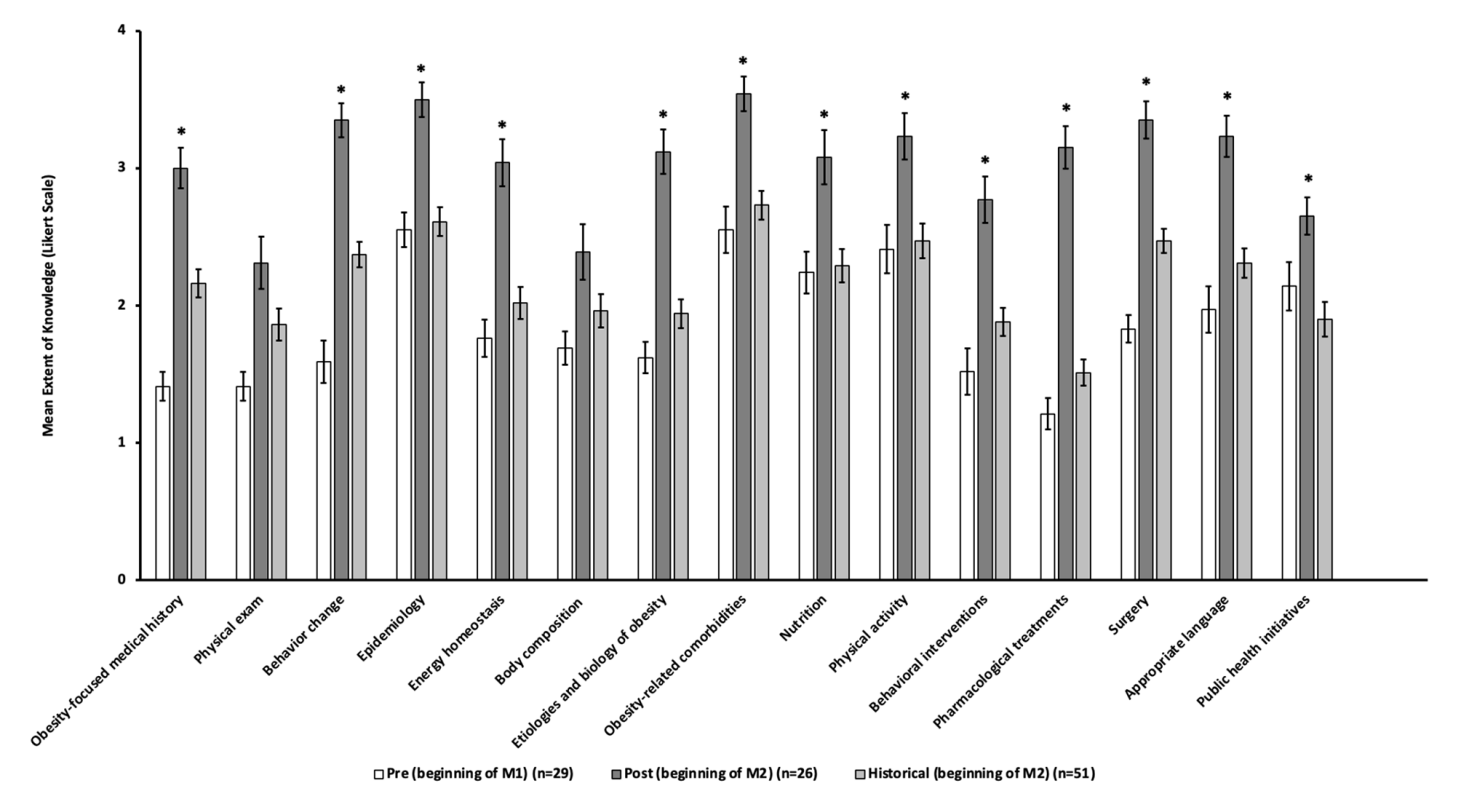
Knowledge of obesity pre- and post-intervention with comparison to historical cohort *post-test vs. historical control *p* < 0.01

After this intervention, knowledge about obesity also improved. Mean scores for knowledge of obesity both before and after the intervention, as well as compared to the historical comparison cohort are presented in Table 4; Fig. 1. Knowledge of obesity in fourteen out of fifteen areas – medical history, physical exam, behavior change, epidemiology, energy homeostasis, body composition, etiologies, comorbidities, nutrition, physical activity, behavioral interventions, pharmacology, surgery, and language – increased significantly from baseline to post-intervention. When comparing the post-intervention group to the historical comparison cohort, obesity knowledge was significantly higher in the post-intervention group in thirteen out of fifteen categories. Knowledge of the physical exam and body composition was not significantly different between the post-intervention group and the historical comparison cohort. The composite knowledge score (out of 60) increased significantly from baseline to post-intervention (27.90 vs. 45.69, *p* < 0.001) and was significantly greater than the historical comparison cohort (32.49, *p* < 0.001).

**Table 4:** Knowledge of obesity*

	Mean (SD) intervention pre-test (beginning of M1) (*n* = 29)	Mean (SD) intervention post-test (beginning of M2) (*n* = 26)	Mean (SD) historical control group (beginning of M2) (*n* = 51)	Pre-test vs. post-test *p*-value	Post-test vs. historical control *p*-value
Obesity-focused medical history	1.41 (0.57)	3.00 (0.75)	2.16 (0.73)	< 0.001	< 0.001
Comprehensive physical exam in patients with obesity	1.41 (0.57)	2.31 (0.97)	1.86 (0.83)	< 0.001**	0.04
Behavior change	1.59 (0.83)	3.35 (0.63)	2.37 (0.66)	< 0.001	< 0.001
Obesity epidemiology	2.55 (0.69)	3.50 (0.65)	2.61 (0.75)	< 0.001	< 0.001
Energy homeostasis	1.76 (0.74)	3.04 (0.87)	2.02 (0.84)	< 0.001	< 0.001
Body composition	1.69 (0.66)	2.39 (1.02)	1.96 (0.87)	< 0.01**	0.06
Etiologies, mechanisms, and biology of obesity	1.62 (0.62)	3.12 (0.82)	1.94 (0.73)	< 0.001	< 0.001
Obesity-related comorbidities	2.55 (0.91)	3.54 (0.65)	2.73 (0.75)	< 0.001	< 0.001
Nutrition interventions	2.24 (0.83)	3.08 (1.02)	2.29 (0.88)	< 0.01	< 0.001
Physical activity interventions	2.41 (0.95)	3.23 (0.86)	2.47 (0.90)	< 0.01	< 0.001
Behavioral interventions	1.52 (0.91)	2.77 (0.86)	1.88 (0.74)	< 0.001	< 0.001
Pharmacological treatments	1.21 (0.62)	3.15 (0.78)	1.51 (0.67)	< 0.001**	< 0.001
Surgical treatments	1.83 (0.54)	3.35 (0.69)	2.47 (0.64)	< 0.001**	< 0.001
Usage of appropriate language	1.97 (0.91)	3.23 (0.77)	2.31 (0.76)	< 0.001	< 0.001
Policies and public health initiatives	2.14 (0.95)	2.65 (0.69)	1.90 (0.90)	0.03**	< 0.001
Composite knowledge score***	27.90 (6.69)	45.69 (8.84)	32.49 (7.26)	< 0.001	< 0.001

*using a Likert scale from 1 (not at all knowledgeable) to 4 (very knowledgeable)

**Welch’s test used instead of Student’s test

***computed by summing the obesity knowledge questions (out of 75)

## Discussion

Obesity is one of the most pressing US public health concerns. Yet there remains a lack of sufficient obesity education in medical training. This curricular improvement project enhanced self-reported attitudes toward and knowledge of obesity among first-year medical students at CWRU, offering a practical mechanism to introduce more obesity education into undergraduate medical curricula.

A 2021 review of the state of obesity education found only 17 high-quality studies since 1982 that have attempted to improve obesity education at the undergraduate medical level, and only seven of these studies included first-year medical students [15]. Based on their review of the 17 studies, the authors suggested several important qualities in any obesity medical education intervention: teaching behavior change techniques, using small group sessions, using in-person learning, teaching obesity pathophysiology, and placing the intervention in the preclinical years of medical school [15]. Our intervention was able to implement all of these recommendations.

This study has several strengths. Our study is the only study to date that evaluates the implementation of a comprehensive obesity preclinical curriculum. The use of a historical control group also improved our ability to make interpretations about the effect of the new curriculum on obesity attitudes and knowledge. There are also several limitations to this present study. First, a new questionnaire was implemented. Although adapted from a validated tool, this questionnaire does not yet have established reliability or validity among this population. This questionnaire also relied on self-reported beliefs and knowledge, which may vary from actual attitudes and knowledge. As this was an optional survey with no incentive, there was a low response rate, which may limit the power to detect statistical significance. In addition, although we were able to verify the implementation of all of the large changes, we were not able to verify the implementation of all of the recommendations (see Table 1). It is possible that a limited number of recommendations were never implemented.

Moving forward, there are several improvements that can be made to this curriculum. First, the curriculum needs to better address both [1] body composition and [2] the physical exam in patients with obesity. Knowledge in both of these areas was not significantly different post-intervention vs. the historical cohort. Second, all recommendations need to be verified and reinforced to ensure the fidelity and sustainability of the curriculum. Finally, the curriculum needs to be extended into the clinical years, in order to continue obesity education throughout all years of medical school.

## Conclusions

This study showed that first-year medical student self-reported attitudes toward obesity and knowledge of obesity significantly improved after a preclinical obesity curriculum was implemented. This curriculum provides a model for other medical schools to follow in improving their own curricula. Making small changes throughout the entire preclinical curriculum is both a practical and innovative route toward increasing obesity education. Improved obesity education at the undergraduate medical level will serve to create a generation of physicians that are more confident and competent in obesity management.

### Electronic supplementary material

Below is the link to the electronic supplementary material.

Supplementary Material 1

## Data Availability

The datasets used and/or analyzed during the current study are available from the corresponding author on reasonable request.

## Abbreviations

OMEC: Obesity Medicine Education Collaborative
CWRU: Case Western Reserve University School of Medicine
