# Supplementary material for: Preclinical obesity curriculum: audit, implementation, and evaluation
Source: BMC Med Educ. 2024 Jun 7;24:639. doi: 10.1186/s12909-024-05606-9 (PMC11157830; doi:10.1186/s12909-024-05606-9)
Supplement: Supplementary file 1 — Supplementary Material 1 [file 12909_2024_5606_MOESM1_ESM.docx]

**Additional File 1: 37-item questionnaire used to assess student obesity attitudes and knowledge**

| **Obesity attitudes** | 1. Obesity is a disease. |
| --- | --- |
|  | 1. Obesity is caused by poor personal choices. |
|  | 1. On average, individuals with obesity have less willpower than individuals without obesity. |
|  | 1. On average, individuals with obesity are more lazy than individuals without obesity. |
|  | 1. On average, individuals with obesity are more emotional than individuals without obesity. |
|  | 1. People can eat a healthy diet if they choose to do so. |
|  | 1. Counseling about nutrition does not change behavior. |
|  | 1. Patients are likely to follow an agreed-upon plan to increase their exercise. |
|  | 1. Even if I counsel them, patients will continue their poor exercise habits. |
|  | 1. Weight loss is the result of eating less and exercising more. |
|  | 1. It is usually sufficient to give a person brief, clear advice about weight management. |
|  | 1. Weight management counseling takes too much time. |
|  | 1. I think patients with obesity are motivated to change their lifestyle. |
|  | 1. I believe that my patients will follow through with a weight management program. |
|  | 1. I believe patients can maintain weight loss. |
|  | 1. Patients know the health risks associated with obesity. |
|  | 1. Patients take their weight seriously. |
|  | 1. I feel confident treating patients with obesity. |
|  | 1. I feel effective in helping patients with obesity manage their weight. |
|  | 1. I think treating patients with obesity is not worth the time. |
|  | 1. If a patient has obesity, I feel uncomfortable discussing their weight. |
|  | 1. I have a personal desire to counsel patients about weight management. |
| **Obesity knowledge** | 1. Knowledge of a comprehensive obesity-focused medical history (including questions on exercise, diet, sleep, stress) from a patient with obesity. |
|  | 1. Knowledge of a comprehensive physical exam (including use of size-appropriate gowns, BP cuffs, etc.) in patients with obesity. |
|  | 1. Knowledge of evidence-based models of behavior change (e.g. motivational interviewing) to effectively assess patients’ readiness for weight management counseling. |
|  | 1. Knowledge of obesity epidemiology (i.e. common environmental, social, and behavioral contributors to the obesity epidemic; incidence and prevalence of obesity, etc.) |
|  | 1. Knowledge of energy homeostasis and weight regulation (i.e. physiology of obesity and related hormones, e.g. ghrelin, leptin, POMC, NPY, etc.) |
|  | 1. Knowledge of body composition measurements (e.g. waist circumference, bioimpedance analysis, waist-to-hip ratio) and clinical assessment tools of energy expenditure (e.g. indirect calorimetry). |
|  | 1. Knowledge of the etiologies, mechanisms, and biology of obesity (including genetic forms of obesity). |
|  | 1. Knowledge of obesity-related comorbidities and corresponding benefits of weight loss. |
|  | 1. Knowledge of nutrition interventions (e.g., calorie balance, macronutrients, energy density) to develop a comprehensive obesity management care plan. |
|  | 1. Knowledge of physical activity (e.g., aerobic, resistance training, exercise prescriptions) to develop a comprehensive obesity management care plan. |
|  | 1. Knowledge of behavioral interventions (e.g., cognitive behavioral therapy) to develop a comprehensive obesity management care plan. |
|  | 1. Knowledge of pharmacological treatments (i.e., recognize the FDA-approved anti-obesity medications as an appropriate form of therapy) as part of a comprehensive obesity management care plan. |
|  | 1. Knowledge of surgical treatments of obesity as part of a comprehensive obesity management care plan. |
|  | 1. Knowledge of appropriate language usage in verbal, non-verbal, and written communication that is non-biased, non-judgmental, respectful, and empathetic when communicating with patients with obesity. |
|  | 1. Knowledge of policies and public health initiatives pertaining to obesity. |
